# Qualitative and quantitative evaluation of computed tomography changes in adults with cystic fibrosis treated with elexacaftor-tezacaftor-ivacaftor: a retrospective observational study

**DOI:** 10.3389/fphar.2023.1245885

**Published:** 2023-09-21

**Authors:** Sabine Dettmer, Oliver Weinheimer, Annette Sauer-Heilborn, Oliver Lammers, Mark O. Wielpütz, Jan Fuge, Tobias Welte, Frank Wacker, Felix C. Ringshausen

**Affiliations:** ^1^ Institute of Diagnostic and Interventional Radiology, Hannover Medical School, Hannover, Germany; ^2^ Biomedical Research in Endstage and Obstructive Lung Disease Hannover (BREATH), Member of the German Center for Lung Research (DZL), Hannover, Germany; ^3^ Department of Diagnostic and Interventional Radiology, Heidelberg University Hospital, Heidelberg, Germany; ^4^ Translational Lung Research Center Heidelberg (TLRC), German Center for Lung Research (DZL), University of Heidelberg, Heidelberg, Germany; ^5^ Department of Respiratory Medicine and Infectious Diseases, Hannover Medical School, Hannover, Germany; ^6^ European Reference Network on Rare and Complex Respiratory Diseases (ERN-LUNG), Frankfurt, Germany

**Keywords:** cystic fibrosis, elexacaftor-tezacaftor-ivacaftor, computed tomography, quantitative CT, CFTR, therapy response

## Abstract

**Introduction:** The availability of highly effective triple *cystic fibrosis transmembrane conductance regulator* (*CFTR*) modulator combination therapy with elexacaftor–tezacaftor–ivacaftor (ETI) has improved pulmonary outcomes and quality of life of people with cystic fibrosis (pwCF). The aim of this study was to assess computed tomography (CT) changes under ETI visually with the Brody score and quantitatively with dedicated software, and to correlate CT measures with parameters of clinical response.

**Methods:** Twenty two adult pwCF with two consecutive CT scans before and after ETI treatment initiation were retrospectively included. CT was assessed visually employing the Brody score and quantitatively by YACTA, a well-evaluated scientific software computing airway dimensions and lung parenchyma with wall percentage (WP), wall thickness (WT), lumen area (LA), bronchiectasis index (BI), lung volume and mean lung density (MLD) as parameters. Changes in CT metrics were evaluated and the visual and quantitative parameters were correlated with each other and with clinical changes in sweat chloride concentration, spirometry [percent predicted of forced expiratory volume in one second (ppFEV_1_)] and body mass index (BMI).

**Results:** The mean (SD) Brody score improved with ETI [55 (12) vs. 38 (15); *p* < 0.001], incl. sub-scores for mucus plugging, peribronchial thickening, and parenchymal changes (all *p* < 0.001), but not for bronchiectasis (*p* = 0.281). Quantitatve WP (*p* < 0.001) and WT (*p* = 0.004) were reduced, conversely LA increased (*p* = 0.003), and BI improved (*p* = 0.012). Lung volume increased (*p* < 0.001), and MLD decreased (*p* < 0.001) through a reduction of ground glass opacity areas (*p* < 0.001). Changes of the Brody score correlated with those of quantitative parameters, exemplarily WT with the sub-score for mucus plugging (r = 0.730, *p* < 0.001) and peribronchial thickening (r = 0.552, *p* = 0.008). Changes of CT parameters correlated with those of clinical response parameters, in particular ppFEV_1_ with the Brody score (r = −0.606, *p* = 0.003) and with WT (r = −0.538, *p* = 0.010).

**Discussion:** Morphological treatment response to ETI can be assessed using the Brody score as well as quantitative CT parameters. Changes in CT correlated with clinical improvements. The quantitative analysis with YACTA proved to be an objective, reproducible and simple method for monitoring lung disease, particularly with regard to future interventional clinical trials.

## 1 Introduction

Cystic fibrosis (CF) is the most common genetic disease in Caucasian populations. It is an autosomal recessive genetic disease with mutations in the gene encoding the CF transmembrane conductance regulator (CFTR) protein (Shteinberg et al., 2021). Life expectancy increased from 1 year in the 1950s to over 50 years nowadays (Bell et al., 2020). Therapy comprises nutritional support, antibiotic therapy, physiotherapy for mucus clearance, mucoactive drugs, and treatment of CF-related complications (Cystic Fibrosis Foundation, 2021). In addition, CFTR modulators had been developed over the past two decades (Bell et al., 2020), with four CFTR modulators and their combinations approved for people with CF (pwCF) and different target mutations and varying eligibility criteria across countries today: ivacaftor in pwCF and gating mutations; lumacaftor-ivacaftor and tezacaftor-ivacaftor in pwCF who are homozygous for the *F508del* mutation; tezacaftor-ivacaftor in pwCF who are heterozygous for *F508del* and a residual function mutation; and elexacaftor-tezacaftor-ivacaftor (ETI) in those with at least one *F508del* mutation (Duckers et al., 2021; Regard et al., 2022). ETI was approved in Europe in late August 2020. CFTR modulators partially restore CFTR protein function with clinical efficacy in terms of lung function, pulmonary exacerbations, nutritional status, quality of life (Burgel et al., 2020; Nichols et al., 2022) and lung morphology (Arnaud et al., 2021; Graeber et al., 2022a; 2022b; Schütz et al., 2023; Stanke et al., 2023).

Imaging is an important noninvasive method to assess CF lung disease. Even though the progression of lung disease is routinely assessed by pulmonary function tests (PFT), chest imaging has been shown to be more sensitive than PFTs in the detection of structural lung damage (de Jong et al., 2004). In contrast to PFT, imaging provides regional information and differentiates between the different components of lung disease, indicating specific complications like mycobacterial disease or (allergic bronchopulmonary) aspergillosis. Computed tomography (CT) is superior to plain chest X-ray in detecting early and subtle pulmonary changes in CF (de Jong et al., 2006; Davis et al., 2007). The advantages of MRI are the functional imaging of ventilation and perfusion and the radiation-free technique. Nevertheless, the availability and expertise is better in CT qualifying CT as the method of choice for imaging of CF in many centers, in particular with special low dose protocols and among adult pwCF and advanced lung disease (Sileo et al., 2014; Robinson et al., 2023). The airways and the lung parenchyma are adequately assessed natively and intravenous contrast medium is reserved for specific indications such as vascular imaging and intervention planning for pulmonary hemorrhage (Wielpütz et al., 2016). Typical imaging findings in CF are bronchiectasis, airway wall thickening, mucus plugging, consolidations and atelectasis. In the very early stages of the disease, only slight density inhomogeneities of the lung parenchyma and air trapping during expiration can be detected. In advanced lung disease, emphysema, bronchiectatic destruction of lobes and dilatation of bronchial arteries with subsequent pulmonary hemorrhage occur, which may require invasive diagnostics and interventional therapy (Loeve et al., 2009).

Various scoring systems for imaging have been developed to objectively and reproducibly assess disease severity and response to treatment (Calder et al., 2014). The most common CT scores are the Bhalla (Bhalla et al., 1991) and the Brody (Brody et al., 2004; 2006) scores. In both, bronchiectasis, peribronchial thickening, mucus plugging and parenchymal abnormalities are evaluated and severity and extent are assessed semi-quantitatively. In MRI, considerable progress has been made in terms of resolution with sequences with a very short echo time, so that visual scores could also be developed for MRI as alternative to CT (Dournes et al., 2021), e.g., the Eichinger and the Helbich scores (Sileo et al., 2014). An MRI score has also been used for evaluation of treatment response to ETI (Graeber et al., 2022a). Limitations of all visual scores are the semi-quantitative assessment, the time consuming evaluation and the reader dependency. However, they are the standard at most centers and imaging response studies to CFTR modulators have been evaluated with visual CT scores (Sheikh et al., 2015; Chassagnon et al., 2016; Arnaud et al., 2021).

A more objective approach to airway assessment has emerged during the last two decades with computer-assisted quantitative analysis of the airways and lung parenchyma. Therefore, different software tools were developed predominantly for scientific purposes (Nakano et al., 2002; Weinheimer et al., 2008; Grydeland et al., 2010). These quantitative tools work in CT, but so far no comparable one could be developed for MRI. Nevertheless, quantitative scores have important advantages over visual scores: the analyses are more objective, allow quantitative measurements and are less time consuming for the radiologist. This is particularly beneficial for clinical trials evaluating the response to therapy of new drugs (Chen et al., 2023).

The aim of this study was to assess the treatment response to ETI in chest CT visually with the Brody score and quantitatively computer-based with the YACTA software and to evaluate correlation between both methods and measures of clinical response.

## 2 Materials and methods

### 2.1 Study design and study population

This retrospective, observational and non-interventional single-centre study was approved by the Internal Review Board (No. 2923–2015) at Hannover Medical School, Germany. We included all adult pwCF and advanced lung CF disease who attended our specialized adult CF clinic between March 2020 and June 2021, initiated ETI treatment within this time period and had received at least two consecutive CT examinations before and after ETI treatment initiation at our Radiology Department for a specific clinical indication. We considered only scans without intravenous contrast. All patients had a confirmed diagnosis of CF, including genetic analysis, as well as optimized treatment for CF and CF-related complications according to European standards of care (Castellani et al., 2018). The patient and the treating physician shared the decision to initiate treatment with ETI, either within a managed access program starting in March 2020 or following market authorization after the broad availability of ETI in Germany in late August 2020. Clinical data was extracted from our electronic clinical database (FileMaker Pro, Claris International Inc., Sunnyvale, California, United States), including age, gender, *CFTR* genotype, comorbidities, percent predicted forced expiratory volume in one second (ppFEV_1_), body mass index (BMI) and sweat chloride concentration before and after ETI initiation. For the purpose of our analysis we arbitrarily considered clinical response to treatment with ETI when we observed a reduction of the sweat chloride concentration >20 mmol/L and/or improvements of ppFEV_1_ >5% and/or BMI ≥1 kg/m^2^.

### 2.2 CT data acquisition

CT scans before the initiation of ETI were performed on a 64 row MDCT (Lightspeed VCT, GE Healthcare, Milwaukee, WI, United States) or a dual source CT (Somatom Force, Siemens, Erlangen, Germany) for the following clinical indications: evaluation for lung transplantation (*n* = 11; 50%); nontuberculous mycobacterial pulmonary disease (*n* = 8; 36%); and allergic bronchopulmonary aspergillosis (*n* = 3; 14%). The CT examinations with ETI were performed on a photon-counting CT scanner (Naetom alpha, Siemens, Erlangen, Germany) for evaluation of treatment response. Data were reconstructed with the Br40 kernel Q3 and a 512-pixel image matrix. A low dose protocol and no intravenous contrast were used. All CT data were acquired volumetrically with a slice thickness of 1.25 mm. Only CT without intravenous contrast were evaluated.

### 2.3 CT data evaluation

Evaluation of CT data was performed visually according to (Brody et al., 2004; 2006) and in addition automatically quantitative. We used the dedicated software YACTA, which allows an automatic segmentation of the lungs and the airways with following analysis of airway dimensions and lung parenchyma for quantitative measurements (Weinheimer et al., 2008; Achenbach et al., 2012; Wielpütz et al., 2013a). Two consecutive CT examinations before (baseline) and after the initiation of ETI treatment (follow-up) were evaluated. Both time points were evaluated in direct comparison but blinded for the actual timing.

#### 2.3.1 Visual evaluation with the brody score

An experienced radiologist in reading and evaluating chest CT (SD, 15 years of experience) conducted the visual evaluation of the CT examinations. All terms were used according to the definition of the Fleischner Society (Hansell et al., 2008). Bronchiectasis was diagnosed according to the criteria described by Naidich et al. (1982) by the presence of one or more of the following criteria: a bronchoarterial ratio >1, a nontapering bronchus, a bronchus visiable within 1 cm pleura surface. Bronchial wall thickening was defined as a bronchial wall thickness >2 mm in the hila, >1 mm in the central portion of the lung and >0.5 mm in the peripheral lung. The peripheral lung was defined as the portion of the lung within 2 cm of the costal or diaphragmatic pleura.

The modified CT scoring system according to Brody was used (Brody et al., 2004; 2006). Therefore, bronchiectasis, mucus plugging, peribronchial thickening and parenchymal changes were evaluated in sub-scores and separately for each lobe (whereas the lingula was regarded as separate lobe (see online supplement 1). Contrary to the Brody score, we did not analyze air trapping (hyperinflation score) separately because most of the CT were not in expiration. All the sub-scores obtained were added together to a total score and then normalized on a scale of 100. The maximum score was theoretically 216 in contrast to the original Brody score, in which a maximum value of 243 can be achieved with the additional hyperinflation score. Because a lobe could have the majority of the lobe involved by more than one of the parenchymal abnormalities (consolidation, cyst formation, and ground glass opacity), the maximum possible score was 180.

#### 2.3.2 Quantitative evaluation with YACTA

The software YACTA (version 2.9.4.53) was used for quantitative analysis of the airway tree and the lung parenchyma (Weinheimer et al., 2008; Achenbach et al., 2012; Wielpütz et al., 2013a; Konietzke et al., 2018; 2020). The software segmented and analyzed the airway tree and individual lobes and measured various parameters. For airway analysis, the number of airways, total diameter (TD), wall thickness (WT), lumen area (LA), wall percentage (WP), bronchiectasis index (BI), and Pi10 (average airway wall thickness of all airways with an inner perimeter of 10 mm) were calculated as previously described (Achenbach et al., 2012; Wielpütz et al., 2013a; Weinheimer et al., 2017; Kahnert et al., 2023; Weinheimer et al., 2023). Measurements were performed generation-based and for statistical analysis, values were averaged for the fourth to the eighth generation (G4-G8). For lung parenchyma analysis, lung volume, mean lung density (MLD), 75th percentile (P75, as a measure for dense parenchyma like consolidations and ground glass opacities), ground glass opacity index (GGOI), high attenuation areas (HAA), and air density in the tracheal lumen were measured as previously described (Wielpütz et al., 2013b; Jobst et al., 2018; Do et al., 2022). For further details, see online supplement 2 and 3.

### 2.4 Statistical analysis

IBM SPSS Statistics (version 28.0, IBM Corp., Armonk, NY, United States) statistical software program was used to analyse the data. Descriptive data were collected and categorical variables are shown as number (n) and percent (%), ordinal data are shown as median with interquartile range (IQR) and metric data as mean with standard deviation (SD). For paired comparison of the two consecutive CT scans the paired Wilcoxon test was used for categorical data and the paired *t*-test for metric data. Differences with *p*-values <0.05 were regarded as statistically significant. We further evaluated whether changes in Brody score differed in distinct patient groups. Therefore, differences between baseline and follow-up of each feature and the sum scores were calculated. Group comparisons were performed using the Mann-Whitney *U* test for continuous variables. We calculated bivariate correlation between the results of the visual and the quantitative evaluation using Spearman’s correlation coefficient, with rho (r) values < 0.3 indicating weak, values of 0.3–0.49 moderate and values > 0.5 strong correlation (Field, A, 2009).

## 3 Results

### 3.1 Study population and clinical response to elexacaftor-tezacaftor-ivacaftor

Overall, 22 adult pwCF with advanced CF lung disease and two consecutive CT scans before and after ETI treatment initiation were included in our study. Mean (SD) age was 33 (9) years, 15 subjects were males (68%). Table 1 shows the patients’ characteristics.

**TABLE 1:** Patient characteristics (N = 22).

Variable		N (%)
Age, years		33 (9)
Sex		
	Male	15 (68)
	Female	7 (32)
CFTR genotype		
	*F508del*/*F508del*	14 (64)
	*F508del*/Minimal function	6 (27)
	*F508del*/Residual function	2 (9)
Comorbidities		
	Pancreatic insufficiency	20 (91)
	CF-related diabetes	9 (41)
	CF-related liver disease	8 (36)
	Allergic bronchopulmonary aspergillosis	9 (41)
	Chronic *P. aeruginosa* infection	9 (41)
	NTM pulmonary disease	9 (41)

Data are shown as numbers (%). Abbreviations: CF, cystic fibrosis; CFTR, cystic fibrosis transmembrane conductance regulator; NTM, nontuberculous mycobacterial.

We evaluated clinical response to ETI, with regard to sweat chloride concentration median 164 (IQR 96–228) days after ETI treatment initiation, while ppFEV_1_ and BMI were reevaluated after median 466 (IQR 360–562) days, close to the time of the CT scan at follow-up (Table 2). In summary, all pwCF showed any clinical response to ETI, as defined above, and continued treatment beyond the study period within shared decision making in clinical care. We observed clinical meaningful and sustained effects with a mean reduction of sweat chloride concentration of −48.7 mmol/L and mean improvements of ppFEV_1_ and BMI of 11.2% and 2.1 kg/m^2^, respectively (Table 2).

**TABLE 2:** Clinical response to ETI treatment (N = 22).

Clinical parameter	Before ETI initiation	After ETI initiation	*p*-value
Sweat chloride, mmol/L	91.7 (12.9)	43.1 (17.0)	<0.001
ppFEV_1_	44.9 (15.9)	56.1 (20.2)	<0.001
BMI, kg/m^2^	20.8 (2.7)	23.0 (3.2)	<0.001

Data are shown as mean (SD). Abbreviations: BMI, body mass index; ETI, elexacaftor-tezacaftor-ivacaftor; ppFEV_1_, percent predicted forced expiratory volume in 1 s.

### 3.2 CT evaluation

The mean (SD) time interval between the baseline CT before and the follow-up CT after the initiation of ETI treatment was 1,066 (501) days. The follow-up CT was performed at a minimum 189 days after start of treatment, with a mean (SD) time interval of 436 (121) days. Mean computed tomography dose index (CTDI) was 2.0 mGy (SD 1.4 mGy) for the baseline CT before and 1.1 mGy (SD 1.5 mGy) for the follow-up CT with ETI. The time gap between the clinical visits, when clinical parameters FEV_1_ and BMI were captured and the CT scans were performed, were median 0.5 days (IQR: 0.0–3.3 days) and 0.0 days (IQR: 0.0–0.0 days) before (baseline) and after the initiation of ETI treatment (follow-up, respectively.

**FIGURE 1:**
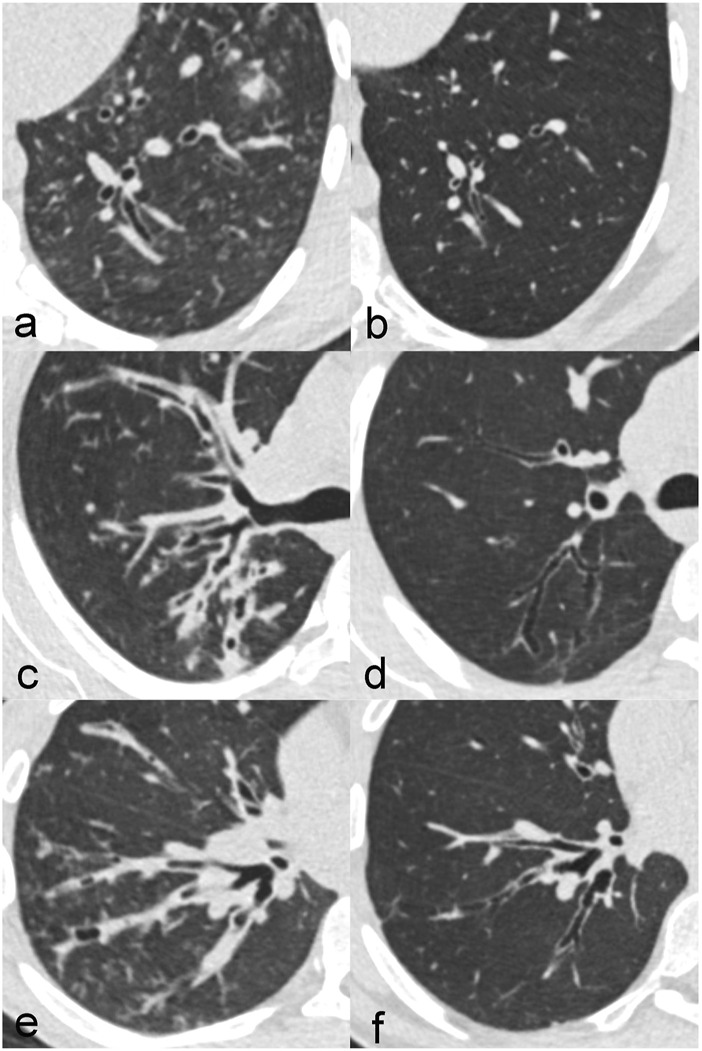
Improvement of CT changes in three different patients with CF between at baseline before treatment **(A,C,E)** and at follow-up after treatment initiation with elexacaftor-tezacaftor-ivacaftor **(B,D,F)**. Patient 1 **(A,B)** is a 20-year old male with impressive regression of bronchiectasis. Patient 2 **(C,D)** is a 24-year old female in which peribronchial thickening clearly decreased. Patient 3 **(E,F)** is a 20-year old male with a reduction of mucus plugging.

CT changes showed an improvement between the baseline CT before and the follow-up CT with ETI treatment. In particular, mucus plugging and peribronchial thickening decreased, but also bronchiectasis showed a partial response (Figure 1).

#### 3.2.1 Visual evaluation with the brody score

Results of the visual analysis with the Brody score are shown in Table 3. We observed significant reductions in the Brody score (from 55 to 38, *p* < 0.001), the mucus plugging sub-score (from 11 to 4, *p* < 0.001), the peribronchial thickening sub-score (from 20 to 13, *p* < 0.001) and the parenchyma sub-score (from 4 to 3, *p* = 0.001). There was a reduction but without statistical significance in the sub-score of bronchiectasis (decrease from 20 to 18, *p* = 0.281).

**TABLE 3:** Results for the Brody score (N = 22).

Score	Before ETI		With ETI		*p*-value
	Median (IQR)	Mean (SD)	Median (IQR)	Mean (SD)	
Bronchiectasis	19.66 (14.72–24.32)	19.67 (5.77)	17.01 (12.85–25.03)	18.24 (7.47)	0.281
Mucus plugging	11.11 (8.88–12.92)	11.04 (2.52)	3.89 (2.78–6.11)	4.39 (2.19)	<0.001
Peribronchial thickening	19.86 (15.00–24.48)	19.91 (5.35)	14.31 (7.22–17.26)	12.78 (6.32)	<0.001
Parenchymal changes	4.17 (2.78–5.00)	4.04 (1.10)	2.78 (1.53–3.89)	2.70 (1.49)	0.001
Total score	57.22 (42.99–63.80)	54.64 (11.92)	38.47 (25.00–48.09)	38.12 (14.66)	<0.001

CT, changes before and with ETI, treatment initiation were compared with Wilcoxon paired test. Abbreviations: ETI, elexacaftor-tezacaftor-ivacaftor; IQR, interquartile range; SD, standard deviation.

#### 3.2.2 Quantitative evaluation with YACTA

Quantitative measurements of airway dimensions revealed significant differences between the baseline CT before and the follow-up CT after ETI treatment initiation (Table 4). There was a significant decrease of WP (from 57% to 49%, *p* > 0.001), Pi10 (from 0.32 mm to 0.26 mm, *p* = 0.004), the WT G4-8 (from 1.60 mm to 1.37 mm, *p* = 0.004) and a significant increase of LA G4-8 (from 20 mm^2^ to 24 mm^2^ (*p* = 0.003), number of segmentable airways (from 122 to 204, *p* = 0.018) and bronchiectasis index (from 2.77 to 7.38, *p* = 0.012).

**TABLE 4:** Group comparisons of airway dimensions ETI treatment initiation (N = 22).

	Before ETI		With ETI		*p*-value
	Mean (SD; variance)	Median (IQR)	Mean (SD; variance)	Median (IQR)	
Number of airways	126.14 (85.66; 7,338.65)	110.00 (69.00–167.75)	203.68 (132.37; 17,521.18)	166.50 (92.50–305.50)	0.018
WP (%)	56.50 (SD 6.68; 44.61)	55.31 (50.52–63.80)	49.41 (6.73; 45.34)	50.34 (44.18–52.77)	<0.001
WT G4-8 (mm)	1.60 (0.35; 0.12)	1.51 (1.30–1.90)	1.37 (0.24; 0.06)	1.32 (1.17–1.65)	0.004
LA G4-8 (mm^2^)	19.52 (5.08; 25.81)	19.77 (15.35–23.02)	24.37 (8.70; 75.77)	21.93 (17.88–30.14)	0.003
Pi10 (cm)	0.32 (0.09; 0.01)	0.31 (0.24–0.40)	0.26 (0.07; 0.01)	0.24 (0.20–0.28)	0.004
BI	2.77 (2.53; 6.38)	1.65 (0.90–5.26)	7.38 (9.79; 95.78)	3.82 (0.55–10.98)	0.012

Group comparisons were performed with dependent *t*-test. Abbreviations: ETI, elexacaftor-tezacaftor-ivacaftor; SD, standard deviation; WT, wall thickness; WP, wall percentage; LA, lumen area; BI, bronchiectasis index; Pi10, standardized measure of the average airway wall thickness of all airways with an inner perimeter of 10 mm; G4-8, average values over the airway generations 4–8.

Quantitative measurements of lung parenchyma revealed significant differences between the baseline CT before and the follow-up CT after ETI treatment initiation (Table 5). In summary, we observed a significant decrease of MLD (from −733 HU to −785 HU, *p* < 0.001) and an increase of lung volume (from 5,635 mL to 6,418 mL, *p* < 0.001). The histogram shifted to the left side with a reduction of P75 (from −730 HU to −813 HU, *p* < 0.001) and a decrease of GGOI (from 14% to 7%, *p* < 0.001) and HAA (from 19% to 14%, *p* < 0.001). In addition, tracheal air density was significant different (−975.88 before versus −1,007.83 after ETI treatment initiation, *p* < 0.001).

**TABLE 5:** Group comparisons of lung parenchyma measures before and with ETI treatment (N = 22).

	Before ETI		With ETI		*p*-value
	Mean (SD; variance)	Median (IQR)	Mean (SD; variance)	Median (IQR)	
Lung Volume (cm^3^)	5,635.40 (1,302.85; 1,697,419.56)	5,364.68 (4,610.67–7,186.23)	6,418.24 (1,190.56; 1,417,435.95)	6,532.13 (5,376.00–7,492.11)	<0.001
MLD (HU)	−732.62 (40.24; 1,619.57)	−735.83 (−771.93 to −712.43)	−784.98 (26.61; 708.26)	−790.05 (−799.79 to −771.79)	<0.001
P75 (HU)	−730.32 (66.17; 4,378.13)	−726.00 (−795.00 to −684.75)	−813.00 (38.61; 1,490.48)	−817.00 (−843.75 to −787.50)	<0.001
GGOI (%)	14.34 (7.69; 59.14)	12.03 (8.61–18.14)	7.01 (3.06; 9.37)	6.64 (4.42–9.14)	<0.001
HAA (%)	19.22 (5.43; 29.44)	17.84 (15.31–21.10)	13.98 (2.18; 4.77)	13.54 (12.66–15.37)	<0.001
Tracheal Air density (HU)	−975.88 (24.48; 599.43)	−987.90 (−991.49 to −947.22)	−1,007.83 (8.22; 67.50)	−1,008.47 (−1,013.27 to −1,006.61)	<0.001

Group comparisons were performed with dependent *t*-test. Abbreviations: ETI, elexacaftor-tezacaftor-ivacaftor; SD, standard deviation; MLD, mean lung density; P15, 15th percentile; GGOI, ground glass opacity index; HU, hounsfield units.

### 3.3 Correlations

Changes between baseline and follow-up CT were calculated. There were moderate and strong correlations between changes in the Brody score and changes in quantitative measurements (Table 6). In particular, we observed strong correlations between the changes of the bronchiectasis sub-score and the number of segmentable airways (r = 0.583, *p* = 0.004) as well as the BI (r = 0.631, *p* = 0.002).

**TABLE 6:** Correlation between changes in visual and quantitative CT parameters (N = 22).

	Bronchiectasis score	Mucus plugging score	Peribronchial thickening score	Parenchyma score	Total score
Number of airways	0.583 (*p* = 0.004)	−0.369 (*p* = 0.091)	−0.141 (*p* = 0.531)	−0.110 (*p* = 0.627)	−0.086 (*p* = 0.702)
WP	−0.287 (*p* = 0.195)	0.617 (*p* = 0.002)	0.401 (*p* = 0.064)	0.114 (*p* = 0.613)	0.361 (*p* = 0.361)
WT G4-8	−0.002 (*p* = 0.994)	0.730 (*p* < 0.001)	0.552 (*p* = 0.008)	0.190 (*p* = 0.398)	0.572 (*p* = 0.005)
LA G4-8	0.451 (*p* = 0.035)	−0.349 (*p* = 0.112)	−0.179 (*p* = 0.425)	−0.013 (*p* = 0.054)	−0.118 (*p* = 0.601)
Pi10	−0.319 (*p* = 0.148)	0.512 (*p* = 0.015)	0.395 (*p* = 0.069)	0.232 (*p* = 0.299)	0.377 (*p* = 0.084)
BI	0.631 (*p* = 0.002)	−0.307 (*p* = 0.176)	−0.025 (*p* = 0.913)	0.135 (*p* = 0.561)	0.043 (*p* = 0.854)
Lung Volume	0.019 (*p* = 0.932)	−0.144 (*p* = 0.523)	−0.485 (*p* = 0.022)	−0.536 (*p* = 0.010)	−0.432 (*p* = 0.045)
MLD	0.086 (*p* = 0.704)	0.432 (*p* = 0.045)	0.722 (*p* < 0.001)	0.491 (*p* = 0.020)	0.663 (*p* < 0.001)
P75	0.073 (*p* = 0.745)	0.417 (*p* = 0.053)	0.700 (*p* < 0.001)	0.481 (*p* = 0.023)	0.626 (*p* = 0.002)
GGOI	0.139 (*p* = 0.537)	0.332 (*p* = 0.131)	0.779 (*p* < 0.001)	0.603 (*p* = 0.003)	0.684 (*p* < 0.001)

Correlations were assessed with Spearman’s correlation coefficients (rho), with values < 0.3 indicating weak, values of 0.3–0.49 moderate and values > 0.5 strong correlation (Field, A, 2009). Abbreviations: WT, wall thickness; WP, wall percentage; LA, lumen area; BI, bronchiectasis index; Pi10, standardized measure of the average airway wall thickness of all airways with an inner perimeter of 10 mm; G4-8, average values over the airway generations 4-8; MLD, mean lung density; P15, 15th percentile; GGOI, ground glass opacity index; HU, hounsfield units.

Furthermore, there were significant correlations between changes of some CT parameters and clinical data (Table 7). Noteworthy is a correlation between ppFEV_1_ and the total Brody score (r = 0.606, *p* = 0.003) as well as WT G4-8 (r = 0.538, *p* = 0.010).

**TABLE 7:** Correlation between quantitative CT and clinical parameters (N = 22).

	BMI, kg/m^2^	ppFEV_1_, %	Sweat chloride, mmol/L
Bronchiectasis score	−0.237 (*p* = 0.288)	−0.332 (*p* = 0.131)	−0.324 (*p* = 0.141)
Mucus plugging score	0.378 (*p* = 0.083)	−0.369 (0.110)	0.153 (*p* = 0.495)
Peribronchial thickening score	−0.356 (*p* = 0.104)	−0.487 (*p* = 0.022)	−0.164 (*p* = 0.467)
Parenchyma Score	−0.358 (*p* = 0.102)	−0.272 (*p* = 0.221)	−0.298 (*p* = 0.178)
Total score	−0.450 (*p* = 0.036)	−0.606 (*p* = 0.003)	−0.165 (*p* = 0.463)
Number of airways	0.243 (*p* = 0.276)	0.046 (*p* = 0.840)	−0.358 (*p* = 0.102)
WP	−0.370 (*p* = 0.090)	−0.344 (*p* = 0.116)	0.406 (*p* = 0.061)
WT G4-8	−0.158 (*p* = 0.482)	−0.538 (*p* = 0.010)	0.020 (*p* = 0.930)
LA G4-8	0.346 (*p* = 0.115)	−0.048 (*p* = 0.832)	−0.370 (*p* = 0.090)
Pi10	−0.195 (*p* = 0.383)	0.243 (*p* = 0.277)	0.160 (*p* = 0.477)
BI	0.201 (*p* = 0.381)	−0.044 (*p* = 0.851)	−0.486 (*p* = 0.025)
Lung Volume	0.162 (*p* = 0.473)	0.063 (*p* = 0.779)	0.216 (*p* = 0.333)
MLD	−0.459 (*p* = 0.032)	−0.165 (*p* = 0.464)	−0.013 (*p* = 0.954)
P75	−0.365 (*p* = 0.095)	−0.184 (*p* = 0.411)	−0.103 (*p* = 0.649)
GGOI	−0.367 (*p* = 0.093)	−0.234 (*p* = 0.294)	−0.137 (*p* = 0.544)

Correlations were assessed with Spearman’s correlation coefficients (rho), with values < 0.3 indicating weak, values of 0.3–0.49 moderate and values > 0.5 strong correlation (Field, A, 2009). Abbreviations: WT, wall thickness; WP, wall percentage; LA, lumen area; BI, bronchiectasis index; Pi10, standardized measure of the average airway wall thickness of all airways with an inner perimeter of 10 mm; G4-8, average values over the airway generations 4–8; MLD, mean lung density; P15, 15th percentile; GGOI, ground glass opacity index; HU, Hounsfield units.

## 4 Discussion

In our study, we demonstrated that the response to treatment with ETI in adult pwCF can be assessed with CT, employing the Brody score as well as a quantitative evaluation with YACTA. CT showed significant improvement in airway and parenchymal parameters between baseline CT before and follow-up CT after ETI treatment initiation. In addition, visual and quantitative CT parameters correlated with each other as well as with clinical measures of treatment response.

The Brody score is a well-established score for visual evaluation of CT changes in patients with CF and it is used for evaluation of disease severity, disease progression and response to therapy. Our results for treatment with ETI are consistent with previous studies previous studies on this topic. The first study evaluating CT changes in patients with ivacaftor monotherapy by Sheikh et al. (2015) showed a significant improvement in the Brody score and its sub-scores for bronchiectasis, mucus plugging and airway wall thickness. While the results for the total score and the mucus plugging and airway wall thickness sub-scores were similar to ours, we could show only insignificant improvement of the bronchiectasis sub-score. However, parenchymal changes were significant improving in our cohort, with a decrease of ground glass opacities and consolidations. Another study by Chassagnon et al. (2016) demonstrated an 11% improvement for total CT score and 32% for mucus plugging after initiation of ivacaftor monotherapy. In contrast to our results, peribronchial thickening did not change significantly and bronchiectasis progressed in this study (Chassagnon et al., 2016). Arnaud et al. (2021) evaluated CT changes under therapy with lumacaftor-ivacaftor with the Brody score. They showed an improvement in total score and mucus plugging sub-score, while peribronchial thickening, bronchiectasis, and parenchymal sub-score did not change. Campredon et al. (2021) investigated the effect of lumacaftor-ivacaftor in CT in a French prospective real-world observational study (Regard et al., 2022). In this large multicenter study, they used the modified Bhalla score for visual assessment of CT. They could show a significant decrease for the Bhalla score related to an improvement of mucus plugging, bronchial wall thickening and parenchymal consolidations. In addition, they performed cluster analysis with machine learning and identified different morphological clusters, which may predict response to treatment. Another artificial intelligence (AI)-based approach was taken by Dournes and coworkers. They validated a fully automated AI-driven scoring system of CF lung disease severity. This allowed fully automated volumetric quantification of CF-related modifications over an entire lung and therefore provides a robust method to quantify disease outcomes and response to treatment for CFTR modulator therapy (Dournes et al., 2022). Studies with treatment response evaluation in MRI showed similar results for semiquantitative analysis with visual scores. Graeber et al. (2022a) evaluated the effects of Elexacaftor/Tezacaftor/Ivacaftor therapy on MRI in patients with cystic fibrosis and one or two F508del alleles. They could demonstrate an improvement of abnormalities in lung morphology, including airway mucus plugging and wall thickening.

For quantitative analysis, we used the YACTA software. This software was developed in the Department of Radiology at Heidelberg University Hospital (Germany); it is well validated and has been used in many previous studies in various lung diseases. Our evaluation with YACTA could confirm treatment response and the results of the semiquantitative evaluation with the Brody score. The number of segmented airways increased significantly with therapy. It can be assumed that a decrease in narrowing and luminal obstruction facilitated the tracking and segmentation of the airways, due to the fact that WT of the airways and mucus plugging decreased. WP is calculated from wall area and lumen area. Its decrease with ETI treatment indicated an increase in lumen and/or a decrease in wall area. Measurements of WT and LA show that it is both, a significant increase in LA and a significant decrease in WT. Again, a reduction in mucus plugging and bronchial wall inflammation in therapy response is the most likely cause. Pi10 decreased significantly between baseline and follow-up. Pi10 represents a standardized measure of the average airway wall thickness in bronchi with 10 mm internal perimeter. It is therefore independent of bronchial generation and facilitates comparability between similarly sized airways. BI is a measure of bronchial narrowing. Its calculation is based on the fact that the bronchial lumen tapers from central to peripheral, and the index is higher the more this is not the case (Weinheimer et al., 2017). The increased bronchiectasis index is surprising. There are two possible explanations: the larger lumen area due to the reduction in mucus plugging and WT and the better segmentation of the bronchi with an increased number for evaluation. The bronchi are still dilated, and the more dilated bronchi can be evaluated the higher the BI is. So, it is probably not the bronchial dilatation that is increasing, but the total number of dilated bronchi. In addition, the larger lumen area due to the reduction in mucus plugging and WT might pretend progressive bronchiectasis. Therefore the evaluation of the total area or the outer diameter would have been more suitable than the lumen area. However, visual analyses with the Brody score revealed that bronchiectasis were not progressive. Quantitative evaluation of lung volume and lung density revealed highly significant changes under treatment with ETI. Of particular note is a significant increase in lung volume and a decrease in lung density. The increase in lung volume is consistent with an improvement in pulmonary function parameters as a result of decreased bronchial obstruction and improved ventilation. For lung density, there is a shift in the density histogram toward more negative values, as evidenced by a decrease in MLD and regions of increased density (GGOI). GGOI are defined as segmented lung voxels ≥−800 HU and <−700 HU, representing the proportion of ground glass opacities (Do et al., 2022). Similarly, the 75th percentile as a measure of dense parenchyma such as consolidations and ground glass opacities shifted to more negative HU values. In addition to lung density measurements, we also evaluated tracheal air density. This can be considered a parameter for the stability and reproducibility of density measurements. Surprisingly, tracheal air density differed significantly between baseline and follow-up. At follow-up examination, CT was performed with a Siemens Naetom Alpha with photoncounting detector compared with a conventional detector at baseline. In addition, a dose reduction was performed with a special low-dose protocol at follow-up (mean CTDI of 2.4 mGy at baseline compared to 1.1 mGy at follow-up CT). Both could be the reason for the observed differences in tracheal air density measurements and are biasing lung parenchymal density measurements. Nevertheless, the differences in measurements (e.g., MLD) are larger than the differences in tracheal air density and visual evaluation revealed similar results, indicating that there might also be a relevant treatment effect on lung density. Intravenous contrast agent is known to have an effect on quantitative measurements; therefore, we excluded CT with contrast from analysis. To our knowledge, there is only one study that quantitatively evaluated CT changes under treatment with CFTR modulators. Lauwers et al. (2021) compared CT scans in patients before and 12 weeks after treatment with lumacaftor and ivacaftor. In this study, CT were assessed with the Brody score and quantitatively with measurements of lobe volume, air trapping, airway volume, airway wall volume, airway resistance, pulmonary blood distribution, ventilation/perfusion matching, internal airflow distribution, and aerosol deposition. They demonstrated a decrease in air trapping and airway wall volume and an improvement in ventilation-perfusion matching. The decrease in airway wall volume is consistent with the decrease in wall thickness and Pi10 in our study and represents an improvement in peribronchial thickening. In contrast to our results, lobar volume after maximal inspiration and airway volume (compared with LA in our study) were not significantly different. The other parameters are not comparable as Lauwers et al. (2021) measured functional parameters such as ventilation and perfusion, whereas we were focusing on bronchial dimensions and lung density parameters.

Evaluation of the response to treatment with ETI using sweat test, lung function and nutritional status showed meaningful and sustained clinical effects, which are in line with the results from the ETI pivotal randomized controlled trials as well as the respective open-label extension study (Heijerman et al., 2019; Middleton et al., 2019; Griese et al., 2021). Correlation analysis examined the relationship between visual and quantitative assessment of CT and clinical parameters of response to treatment with ETI. There were moderate to high correlations between visual and automated quantitative evaluation. The bronchiectasis sub-score correlated with the number of airways and the BI, while the mucus plugging sub-score correlated with WP, WT and Pi10. This reveals that both, the visual as well as the quantitative CT evaluation, could be used equally for evaluation of treatment response to ETI. For changes of imaging and clinical parameters, we found the strongest correlations for ppFEV_1_ with the Brody score and WT. In a previous study, Sheikh et al. also found correlations between the Brody score and lung function parameters for the total score and sub-scores for airway wall thickening and bronchiectasis (Sheikh et al., 2015). In their study with lumacaftor-ivacaftor, Arnaud and others investigated the correlation between clinical parameters and the Brody score (Arnaud et al., 2021). In agreement with our results, they found a significant correlation between the improvement of lung function and the improvement of the total score, the peribronchial thickening sub-score and the mucus plugging sub-score.

Our real-life study has inherent limitations. First, the number of included patients is rather small. However, as ETI has only recently been approved in Germany, there are currently few data available, especially regarding the radiological evaluation of treatment response. Another important limitation is the use of different CT protocols at baseline and follow-up. However, we minimized this bias by excluding contrast-enhanced examinations from quantitative analysis. Nevertheless, the increasing use of low-dose scans and the new photon counting detector at follow-up may have biased the quantitative measurements.

In conclusion, we were able to visualize and quantify the response to treatment with ETI in adult pwCF on CT. The Brody score improved significantly with treatment and the changes correlate with clinical parameters, which is consistent with previously published studies. In addition, we performed a quantitative analysis with measurements of bronchial dimensions and lung parenchymal density, which revealed a decrease in WT, an increase in LA and segmented airways and a decline of inflammation with decreasing lung density and ground glass opacities. Changes in these quantitative data correlate with the Brody score and improvement in lung function, making this automated measurement an objective, reproducible, and simple method for assessing response to treatment, particularly with regard to future clinical trials.

## Data Availability

The original contributions presented in the study are included in the article/Supplementary Material, further inquiries can be directed to the corresponding author.

## Abbreviations

BI, bronchiectasis index; BL, baseline; BMI, body mass index; CF, cystic fibrosis; CFTR, cystic fibrosis transmembrane conductance regulator; CT, computed tomography; CTDI, computed tomography dose index; ETI, elexacaftor–tezacaftor–ivacaftor; G4-8, average values over the airway generations 4–8; GGOI, ground glass opacitiy index; HU, Hounsfield units; IQR, interquartile range; LA, lumen area; MLD, mean lung density; MRI, magnetic resonance imaging; P75, 75th percentile; PFT, pulmonary function tests; Pi10, standardized measure of the average airway wall thickness of all airways with an inner perimeter of 10 mm; ppFEV_1_, percent predicted forced expiratory volume in one second; pwCF, people with cystic fibrosis; r, rho; SD, standard deviation; TD, total diameter; WT, wall thickness; WP, wall percentage.
