# Supplementary material for: Qualitative and quantitative evaluation of computed tomography changes in adults with cystic fibrosis treated with elexacaftor-tezacaftor-ivacaftor: a retrospective observational study
Source: Front Pharmacol. 2023 Sep 21;14:1245885. doi: 10.3389/fphar.2023.1245885 (PMC10552920; doi:10.3389/fphar.2023.1245885)
Supplement: Supplementary file 1 [file DataSheet1.docx]

**Supplementary Material**

**Qualitative and quantitative evaluation of computed tomography changes in adults with cystic fibrosis treated with elexacaftor-tezacaftor-ivacaftor: a retrospective observational study**

Sabine Dettmer*, Oliver Weinheimer, Annette Sauer-Heilborn, Oliver Lammers, Mark O. Wielpütz, Jan Fuge, Tobias Welte, Frank Wacker, Felix C. Ringshausen

* Correspondence: Corresponding Author: dettmer.sabine@mh-hannover.de

**Supplementary Data 1: Visual evaluation with the Brody score**

The modified HRCT scoring system according to Brody was used for the visual evaluation of CT (Brody et al., 2004, 2006). Therefore, bronchiectasis, mucus plugging, peribronchial thickening and parenchymal changes were evaluated separately for each lobe, whereas the lingula was regarded as separate lobe. Contrary to the Brody score, we did not analyze air trapping separately because most of the CT were not in expiration. For the bronchiectasis score (range 0-12), the extent of bronchiectasis was evaluated on a scale from 0 to 3 (0 = none; 1 = less than 1/3 of the lobe; 2 = 1/3 – 2/3 of the lobe; and 3 = more than 2/3 of the lobe) separately for the central and in the peripheral lung. The sum of both was multiplicated with the average multiplier size (0.5 = 0; 1 = 1; 1.5 = 1.25; 2.0 = 1.5; 2.5 = 1.75 and 3 = 2). The multiplier size was calculated by the sum of the largest dilated bronchus (1 ≤ 2x; 2 = 2x-3x; 3 ≥ 3x the size of the accompanying vessel) and the average size of dilated bronchi (1 ≤ 2x; 2 = 2x-3x; 3 ≥ 3x the size of the accompanying vessel) divides by 2. For the mucus plugging score (range 0-6), central mucus plugging was defined as a plug / an opacity definable in a bronchus, and peripheral mucus plugging was defined as either dilated mucus filled peripheral bronchi or thin branching structures with centrilobular nodules in the peripheral lung (tree in bud pattern). For the peribronchial thickening score (0-9), the extent of peribronchial thickening was evaluated on a scale from 0 to 3 (0 = none; 1 = less than 1/3 of the lobe; 2 = 1/3 – 2/3 of the lobe; and 3 = more than 2/3 of the lobe) separately for the central and in the peripheral lung. The sum of both was multiplicated with the severity of peribronchial thickening (1 = mild; 1.25 = moderate; 1.5 = severe). For the parenchyma score (range 0-9), the extent of dense parenchymal opacities, the extent of ground glass opacities and the extent of cysts were evaluated (0 = none; 1 = less than 1/3 of the lobe; 2 = 1/3 – 2/3 of the lobe; and 3 = more than 2/3 of the lobe) and added to a total score. A score was calculated for each abnormality and summed up to a total score for disease severity in each lobe. The scores for the six lobes were added to provide a total patient score. The maximum score was 216 in contrast to the original Brody score, in which a maximum value of 243 can be achieved with the additional hyperinflation score. Because a lobe could have the majority of the lobe involved by more than one of the parenchymal abnormalities (consolidation, cyst formation, and ground glass opacity), the maximum possible score was 180. All the sub-scores obtained were added together to a total score and then normalized on a scale of 100.

**Supplementary Data 2: Quantitative evaluation with YACTA**

The software YACTA (version 2.9.4.53) was used for quantitative analyzation of the airway tree and the lung parenchyma (Weinheimer et al., 2008; Achenbach et al., 2012; Wielpütz et al., 2013a). The software segmented and analyzed the airway tree, vessels, lungs and individual lobes fully automated without necessity of user interaction or manual correction. Various parameters were analyzed which can be roughly divided into analysis of airways and lung parenchyma.

Airway analysis:

- Number of airways as the sum of all segmented bronchi.
- As measures of airway geometry, total diameter (TD), wall thickness (WT), lumen area (LA), and wall percentage (WP = 100*WA/(LA + WA) were calculated by using the parameter-free integral-based method (Achenbach et al., 2004; Weinheimer et al., 2008). Measurements were performed generation-based in the trachea (G1), main stem (G2), lobar (G3), segmental (G4), and subsegmental (G5–10). For statistical analysis values were averaged for G4-G8.
- Bronchiectasis index (BI) is a measure for bronchial tapering; the calculation is based on the fact that the bronchial lumen decreases from central to peripheral. The index is calculated based on a comparison of bronchial diameter in different bronchial generations. It is higher the more often a peripheral bronchus is larger than the more proximal section (Weinheimer et al., 2017).
- Pi10 represents a standardized measure of the average airway wall thickness. It is determined by plotting the square root of airway wall area against the inner perimeter of the airway for each airway location. Regression analysis is used to determine the square root of the wall area for a hypothetical airway with 10-mm internal perimeter (Grydeland et al., 2010; Jobst et al., 2019; Kahnert et al., 2023).

Lung parenchyma analysis:

- Histograms of the lung parenchyma with exclusion of large airways and vessels were determined for the whole lung and separately for each lobe (Wielpütz et al., 2013b; Jobst et al., 2018).
- Lung volume and estimated lung weight (based on the lung volume and the lung density) were calculated.
- Air density in the tracheal lumen was measured.
- Histogram analysis was performed. Mean lung density (MLD) defined as the average CT value of all segmented lung voxels and percentiles defined as the CT value less than or equal the respective percentage of the lung (with the 15^th^ percentile P15 as a measure for emphysema and the 75^th^ percentile P75 as a measure for dense parenchyma like consolidations and ground glass opacities) were calculated (Wielpütz et al., 2013b; Jobst et al., 2018).
- Empysema volume was calculated based on a threshold of -950 HU and given in ml and percentage of the whole lung volume (emphysema index, EI).
- High attenuation areas (HAA) defined as the percentage of segmented lung voxels ≥-700 HU representing the proportion of consolidations and Ground glass opacity index (GGOI) defined as the percentage of the segmented lung voxels ≥-800 HU and <-700 HU representing the proportion of GGO regions (Do et al., 2022).

**Supplementary Data 3: Illustration of segmentation and analysis of airways and lung parenchyma with YACTA**

**
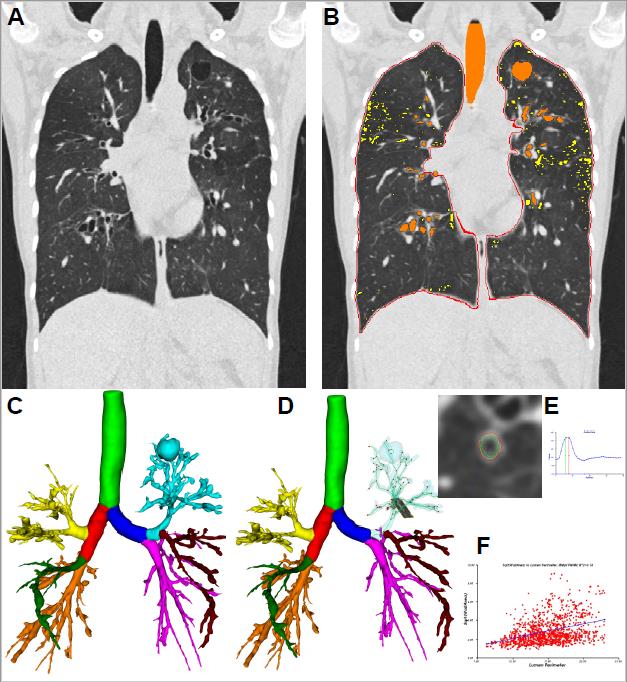
**

**Figure:** Illustration of segmentation and analysis of airways and lung parenchyma. Coronal view of the original image (a) with the segmented lung (red), bronchial tree (orange) and emphysema (yellow) (b). 3D-view of the segmented bronchial tree and in different colors for the trachea and each lung lobe (c) and illustration of the measurements (d-f).
